# Relationship between low molecular weight heparin calcium therapy and prognosis in severe acute kidney injury in sepsis: Mendelian randomized analysis and retrospective study

**DOI:** 10.3389/fphar.2024.1389354

**Published:** 2024-06-10

**Authors:** Jian-Chun Li, Shi-Tao Huang, Fei Feng, Lin-Jun Wang, Ting-Ting Chen, Min Li, Li-Ping Liu

**Affiliations:** ^1^ The First Clinical Medical College of Lanzhou University, Lanzhou, Gansu, China; ^2^ Department of Emergency Critical Care Medicine, The First Hospital of Lanzhou University, Lanzhou, Gansu, China

**Keywords:** sepsis associated acute injury injury, acute injury injury, sepsis, heparanase, heparin-binding protein, Mendelian randomization, genome-wide association studies

## Abstract

**Background:**

Sepsis-associated acute kidney injury (SA-AKI) poses an independent risk for mortality due to the absence of highly sensitive biomarkers and a specific treatment plan.

**Objective:**

Investigate the association between low molecular weight heparin (LMWH) calcium therapy and prognosis in critically ill SA-AKI patients, and assess the causal relationship through Mendelian randomization (MR) analysis.

**Methods:**

A single-center, retrospective, cross-sectional study included 90 SA-AKI patients and 30 septic patients without acute kidney injury (AKI) from the intensive care unit (ICU) of the First Hospital of Lanzhou University. SA-AKI patients were categorized into control or LMWH groups based on LMWH calcium usage. Primary outcome was renal function recovery, with secondary outcomes including 28-day mortality, ICU stay length, number of renal replacement therapy (RRT) recipients, and 90-day survival. MR and related sensitivity analyses explored causal effects.

**Results:**

The combination of heparin-binding protein (HBP), heparanase (HPA), and neutrophil gelatinase-associated lipocalin (NGAL) demonstrated high diagnostic value for SA-AKI. MR analysis suggested a potential causal link between gene-predicted HBP and AKI (OR: 1.369, 95%CI: 1.040–1.801, *p* = 0.024). In the retrospective study, LMWH-treated patients exhibited improved renal function, reduced levels of HPA, HBP, Syndecan-1, and inflammation, along with enhanced immune function compared to controls. However, LMWH did not impact 28-day mortality, 90-day survival, or ICU stay length.

**Conclusion:**

LMWH could enhance renal function in SA-AKI patients. MR analysis supports this causal link, underscoring the need for further validation in randomized controlled trials.

## 1 Background

Sepsis associated acute kidney injury (SA-AKI) is a prevalent and severe complication among critically ill patients, contributing to elevated morbidity and mortality rates (Peerapornratana et al., 2019). Notably, the mortality rate in SA-AKI patients significantly exceeds that of sepsis patients without acute kidney injury (AKI) (Bagshaw et al., 2007). This heightened mortality is attributed to the unclear pathogenesis of SA-AKI, the absence of highly sensitive and specific biomarkers for early diagnosis, and the lack of effective, specific treatment modalities (Peerapornratana et al., 2019). Presently, neutrophil gelatinase-associated lipocalin (NGAL) stands as the most extensively studied biomarker for SA-AKI (Shang and Wang, 2017). Despite NGAL’s potential in SA-AKI diagnosis and prediction, prior studies have indicated that the combination of multiple biomarkers yields superior effects and predictive value compared to a single biomarker (Grover et al., 2014). Further investigation is needed to explore whether biomarkers with heightened sensitivity and specificity or combined detection can enable earlier SA-AKI diagnosis.

Fibroblast growth factors (FGF), also recognized as heparin-binding growth factors (HBF), comprise a protein family consisting of approximately two dozen heparin-binding proteins (HBP) (Ornitz and Itoh, 2001), including aFGF and bFGF. Within the FGF family, proteins signal through at least one of four tyrosine kinase receptors and interact with heparan sulfate proteoglycans (HSPGs). In adults, FGFs serve as homeostatic factors involved in tissue repair and responses to injury (Beenken and Mohammadi, 2009). Previous studies have demonstrated that HBP predicts septic infection-induced organ dysfunction and disease severity. Tapper, H., et al. (Tapper et al., 2002) emphasized the predictive value of HBP for SA-AKI. HBP emerges as a pathogenic biomarker and a potential target for heparin treatment of human SA-AKI (Lee et al., 2012). Heparin inhibits HBP-induced inflammation and reduces interleukin (IL) −6 production (Lee et al., 2012). Studies have indicated that low molecular weight heparin (LMWH) blocks HBP-induced inflammation in renal tubular cells, suggesting a potential avenue for SA-AKI treatment (Fisher et al., 2017).

Studies have indicated that heparanase (HPA) mediates septic AKI (Chen et al., 2017). HPA disrupts the structural integrity of extracellular matrix (ECM) and basement membrane, releasing and activating active substances attached to side chains (Lerolle et al., 2010; Teixeira and Götte, 2020), which leads to the release of heparin-binding molecules promoting pro-inflammatory factors such as IL-2, IL-8, bFGF, and transforming growth factor (TGF)-β within the ECM. These inflammatory factors regulate the interaction between the surface of white blood cells (WBC) and endothelial cells, thereby affecting the recruitment, migration and extravasation of WBC (Carter et al., 2003; Axelsson et al., 2012; Higashi et al., 2020). Heparin can alleviate SA-AKI by inhibiting HPA activity (Hirsh and Levine, 1992; Chen et al., 2015).LMWH has been shown to inhibit HPA-mediated HS degradation (Achour et al., 2016). Therefore, it is speculated that LMWH may inhibit HPA activity, reduce the degradation of ECM, decrease the release of HBP, and improve the renal function and clinical prognosis of SA-AKI.

## 2 Materials and methods

### 2.1 Study design and participants

This retrospective study was conducted in the Department of Critical Care Medicine at the First Hospital of Lanzhou University. Data were collected from patients admitted for sepsis between September 2021 and August 2023 for analysis. The patient population consisted of individuals of Asian descent. Ethics approval for this study was obtained from the Ethics Committee of the First Hospital of Lanzhou University, and informed consent was obtained from the patient or their family.

All participants included in this study met the diagnostic criteria for sepsis-3 (Singer et al., 2016), which entail a positive or suspected infection and a Sequential Organ Failure Assessment (SOFA) score of 2 or higher. The exclusion criteria were as follows (Peerapornratana et al., 2019): age <18 years (Bagshaw et al., 2007), pregnancy or lactation (Shang and Wang, 2017), previous renal underlying diseases (nephrotic syndrome, lupus nephritis, interstitial nephritis, end-stage renal disease, etc.) and a history of kidney transplantation (Grover et al., 2014), malignant tumors or blood system diseases (Ornitz and Itoh, 2001), obstructive urinary tract disease (Beenken and Mohammadi, 2009), expected survival less than 24 h (Tapper et al., 2002), receiving high-dose anticoagulant therapy for conditions such as deep vein thrombosis, atrial fibrillation, or pulmonary embolism (Lee et al., 2012), life-threatening severe organ failure, and (Fisher et al., 2017) discontinuation of treatment requested by a family member.

#### 2.1.1 Group and treatment

**FIGURE 1:**
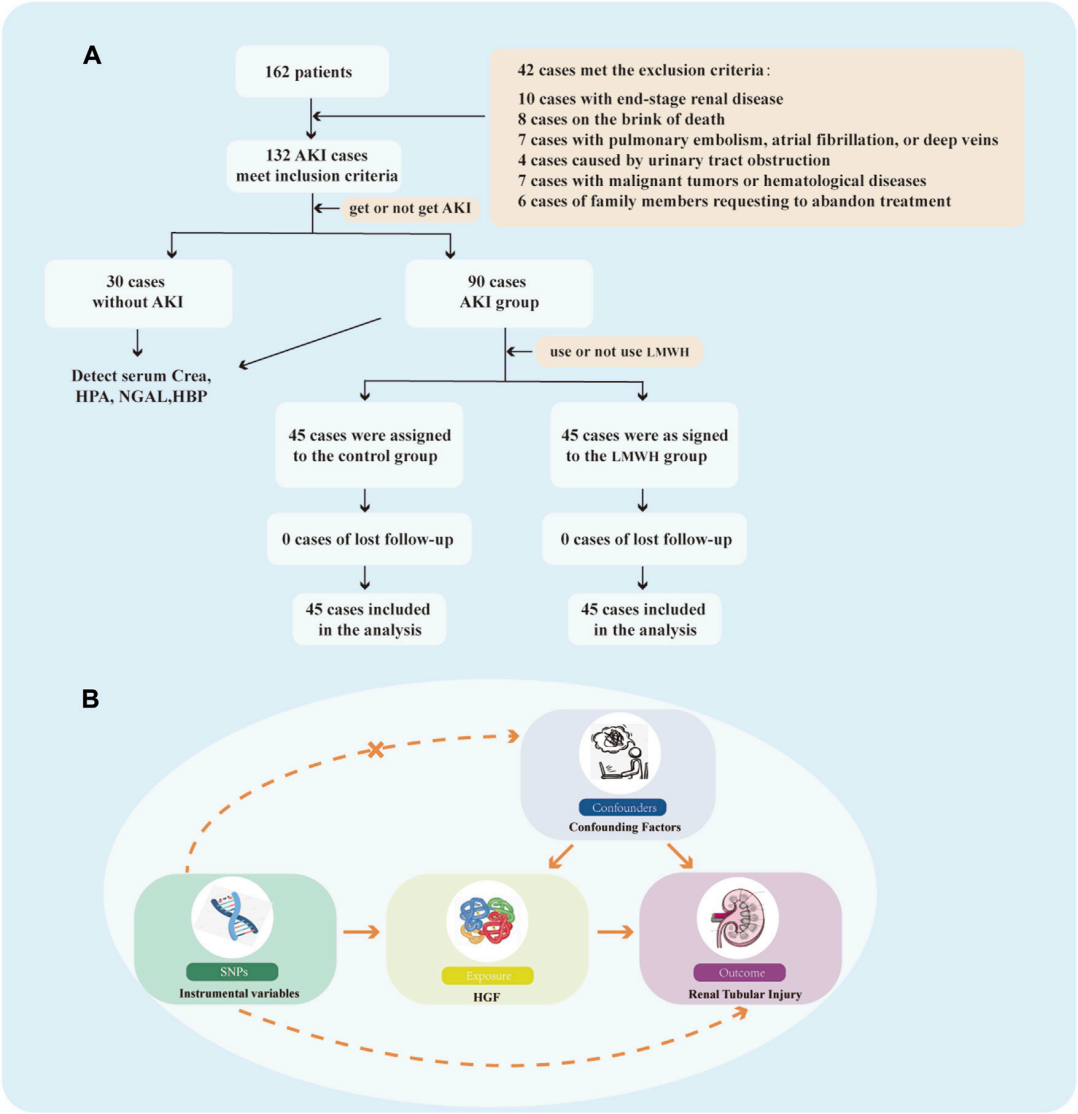
Flow Chart. **(A)** Flow chart illustrating the selection process for patients meeting the inclusion/exclusion criteria. **(B)** Study design of MR. MR, Mendelian randomizatio; SNPs, single nucleotide polymorphisms; HGF, heparin-binding growth factors.

Patients were categorized into septic patients without AKI and SA-AKI groups based on the presence of AKI. Septic patients without AKI were defined as those who did not experience AKI in the first 7 days of sepsis, whereas patients with SA-AKI had already developed AKI at the time of enrolment. SA-AKI was defined by a sudden and sustained decline in renal function with an absolute increase in serum creatinine of ≥0.3 mg/dL (or ≥26.5 µmol/L); or an increase in serum creatinine to more than 1.5 times the baseline value within the previous 7 days; or urine volume <0.5 mL/kg·h for 6 h. SA-AKI, further divided into control and LMWH groups based on LMWH usage (Figure 1A), involved the subcutaneous injection of 5,000 IU of LMWH calcium once a day in the LMWH group. Both groups received additional treatments in accordance with the sepsis-3 criteria and the 2021 KDIGO Clinical Practice Guidelines (Singer et al., 2016; Rovin et al., 2021).

### 2.2 Sample and laboratory analysis

Blood samples were collected within the initial 24 h following study enrollment and subsequently at the 1st, 3rd, and 7th days after LMWH treatment initiation. Plasma processing occurred within 30 min of blood collection, and the samples were stored at −80°C, with precautions taken to prevent repeated freeze-thaw cycles. Measurement of plasma NGAL, HBP, HPA, and syndecan-1 was performed using enzyme-linked immunosorbent assay (ELISA).

### 2.3 Clinical data collection

All clinical data were extracted from medical records, encompassing demographics (age, sex, height, weight, blood pressure, SOFA score, Acute Physiology and Chronic Health Evaluation II (APACHE II) score), renal function, urine volume, routine blood parameters, coagulation function, length of ICU stay, use of renal replacement therapy (RRT), 28-day fatality rate, 90-day survival rate, and other relevant information.

### 2.4 Mendelian randomization method

#### 2.4.1 Design of Mendelian randomization method in this study

The flow diagram of our Mendelian randomization (MR) method is depicted in Figure 1B. Our MR analysis adhered rigorously to the principles outlined in the 2023 STROBE-MR Guidelines by Stephen et al. (Burgess et al., 2019) and the Epidemiological Observation Study report (von Elm et al., 2007). Additionally, our MR study was designed to fulfill three key assumptions (Peerapornratana et al., 2019): Screened instrumental variables (IVs) exhibit a robust association with the exposure (Bagshaw et al., 2007); IVs are unrelated to confounding factors (Shang and Wang, 2017); IVs can solely influence the outcome through the exposure and do not have a direct correlation with the outcome.

#### 2.4.2 Exposure and outcome data sources

Our data were sourced from publicly available genome-wide association studies (GWAS) data repositories. HBF was designated as the exposure and obtained from the IEU Open GWAS project (https://gwas.mrcieu.ac.uk) under catalog number ebi-a-GCST90012067. In the study conducted by Lasse et al. (Folkersen et al., 2020), the total sample size comprised 21,758 individuals, with 1,366,246 single nucleotide polymorphisms (SNPs). Renal tubular injury served as the outcome, and the data were retrieved from FINNGEN database, encompassing a total sample size of 340209 individuals, identified by the catalog number finngen_R8_N14_DISIMPAIRRENTUB. FINNGEN is a substantial GWAS database and analyzes genomic and health data from approximately 500,000 biobank participants from Finland (Locke et al., 2019).

#### 2.4.3 Selection of instrumental variable

Initially, we selected SNPs with a significance threshold of *p* < 5 × 10^6^ from the GWAS as IVs for the exposure. These SNPs were also ensured to be independent, i.e., in linkage disequilibrium (LD) with an R^2^ < 0.001 and LD > 10000 kb. Subsequently, we merged the extracted exposure and outcome data, while the merged dataset was harmonise to ensure that the effects of SNPs on exposure and outcome corresponded to the same alleles (Supplementary Table S1). Additionally, we calculated the R^2^ and F-value of the screened SNPs. An F-statistics exceeding 10 indicates a strong association (Burgess et al., 2013). This calculation is performed using the formula: F = R^2^ (N-K-1)/(1-R^2^) (Palmer et al., 2012), where R^2^ represents the variance in exposure explained by the selected SNPs and N represents the number of genetic samples for the phenotype.

#### 2.4.4 MR and sensitivity analysis

In this study, the primary analysis method employed was the standard inverse variance weighting (IVW) method. Additionally, the MR-Egger method, weighted median method, and maximum likelihood method were used as supplementary analyses to assess the causal relationship between exposure and outcome. The MR analysis was conducted using R software (version 4.3.1), incorporating Two SampleMR (version 0.5.7) and MRPRESSO (version 1.0). A significance level of *p* < 0.05 was considered statistically significant.

To enhance the robustness of the results, a sensitivity analysis was conducted. Heterogeneity was assessed using the Cochran Q test, and horizontal pleiotropy was examined using the MR-Egger intercept test. Leave-one analysis was utilized to investigate the influence of individual SNPs on MR estimates. Additionally, scatter plots and funnel plots were employed to illustrate the results, aiding in the identification of outliers and pleiotropy. The MR-PRESSO method was then employed to identify and address outlier SNPs (Verbanck et al., 2018). To determine causal directionality, we applied the Steiger test for validation, mitigating bias induced by reverse causality (Hemani et al., 2017).

#### 2.4.5 ELISA

Serum samples were appropriately diluted, and standard working solutions were prepared according to the manufacturer’s instructions (Elabscience, Shanghai, China). Separate wells were designated for standards, blanks, and samples; 100 μL of standard, standard and sample diluent, and the serum sample to be tested were added to their respective wells. The plates were then incubated at 37°C for 90 min. Subsequent steps, including the addition of biotinylated antibody working solution, enzyme-binding working solution, substrate solution, and termination solution, were carried out according to the manufacturer’s instructions. Following the reaction, the optical density (OD) of each well was measured at a wavelength of 450 nm using a microplate reader.

#### 2.4.6 Flow cytometry

Flow cytometry analysis was conducted using fluorescein isothiocyanate-labelled mouse anti-human CD3 antibody (2 μL), allophycocyanin-labelled mouse anti-human CD4 antibody (1 μL), and PerCP/Cy5.5 mouse anti-CD8b monoclonal antibody (1 μL). Flow cytometry tubes were prepared by adding these antibodies. A 100-μL aliquot of whole blood obtained from the patient’s peripheral vein was collected and mixed with shaking, followed by incubation at room temperature for 15 min. To the sample, 500 μL of haemolysin, 200 μL of phosphate-buffered saline, and 100 μL of well-mixed microspheres were added. The samples were then subjected to flow cytometry. CD4^+^ and CD8^+^ T cell counts were obtained using Kaluza Analysis software.

#### 2.4.7 Statistical analysis

Statistical analysis was conducted using GraphPad Prism 9 Software (GraphPad Software; San Diego, CA, United States) and SPSS V25 (IBM; Armonk, NY, United States). Data are presented as mean ± standard deviation (SD) if they are normally distributed and as interquartile range (IQR) if they are not. For normally distributed data, *t*-test was used, while for count data not conforming to normal distribution, non-parametric test was used. Specifically, Mann-Whitney test was employed for comparison between the two groups. Correlation analysis was performed using a simple linear regression. Sensitivity and specificity were analyzed by receiver operating characteristic (ROC) curve. Survival curves were compared using log-rank (Mantel-Cox) test. *p* < 0.05 was statistically significant.

## 3 Results

### 3.1 Baseline characteristics of the study population

A total of 162 septic patients participated in this study, comprising 30 patients without AKI and 132 patients with AKI. However, 42 patients were excluded from the analysis due to meeting the exclusion criteria of SA-AKI. Consequently, 90 SA-AKI patients were allocated to either the control group or the LMWH group according to whether LMWH was used or not (Figure 1A). Baseline data for both groups were presented in Table 1.

**TABLE 1:** Characteristics of the Patients with sepsis at Baseline. (Mean ± SD).

Parameter	AKI (*n* = 90)	Without AKI (*n* = 30)	*p*-value
Age (years)	63.93 ± 15.28	62.53 ± 12.55	0.493
Sex-no. (%)			0.317
Male	61 (67.78)	19 (63.33)	
Female	29 (32.22)	11 (36.67)	
BMI (kg/m^2^)	23.81 ± 4.07	23.18 ± 3.22	0.590
MAP (mmHg)	103.81 ± 65.02	101.77 ± 20.30	0.354
Serum creatinine (umol/L)	247.34 ± 89.22	81.77 ± 13.41	<0.001
Plasma HPA (ng/mL)	11.69 ± 2.23	10.57 ± 1.47	0.031
Plasma NGAL (pg/mL)	274.54 ± 59.68	213.63 ± 32.57	0.001
Plasma HBP (ng/mL)	93.94 ± 23.75	72.91 ± 17.99	<0.001
SOFA score^a^	9.41 ± 1.84	9.20 ± 1.79	0.478

Abbreviations: AKI, acute kidney injury; BMI, body mass index; MAP, mean arterial pressure; HPA, heparanase; NGAL, neutrophil gelatinase-associated lipids; HBP, heparin-binding protein.

^a^ The Sequential Organ Failure Assessment (SOFA) score ranges from 0 to 24, with higher scores indicating greater severity of organ dysfunction. *p* < 0.05 was statistically significant.

There were no significant differences noted in age, sex, body mass index (BMI), or SOFA scores between the septic patients without AKI and those with SA-AKI. However, notable disparities existed in plasma concentrations of NGAL, HPA, and HBP (*p* < 0.05). Particularly, the concentration of HPA in the SA-AKI group was significantly higher than that in the patients without AKI group.

### 3.2 The combination of HPA, HBP and NGAL had more predictive value in the diagnosis of SA-AKI

#### 3.2.1 Plasma HPA concentration was positively correlated with NGAL, HBP, syndecan-1 and creatinine

**FIGURE 2:**
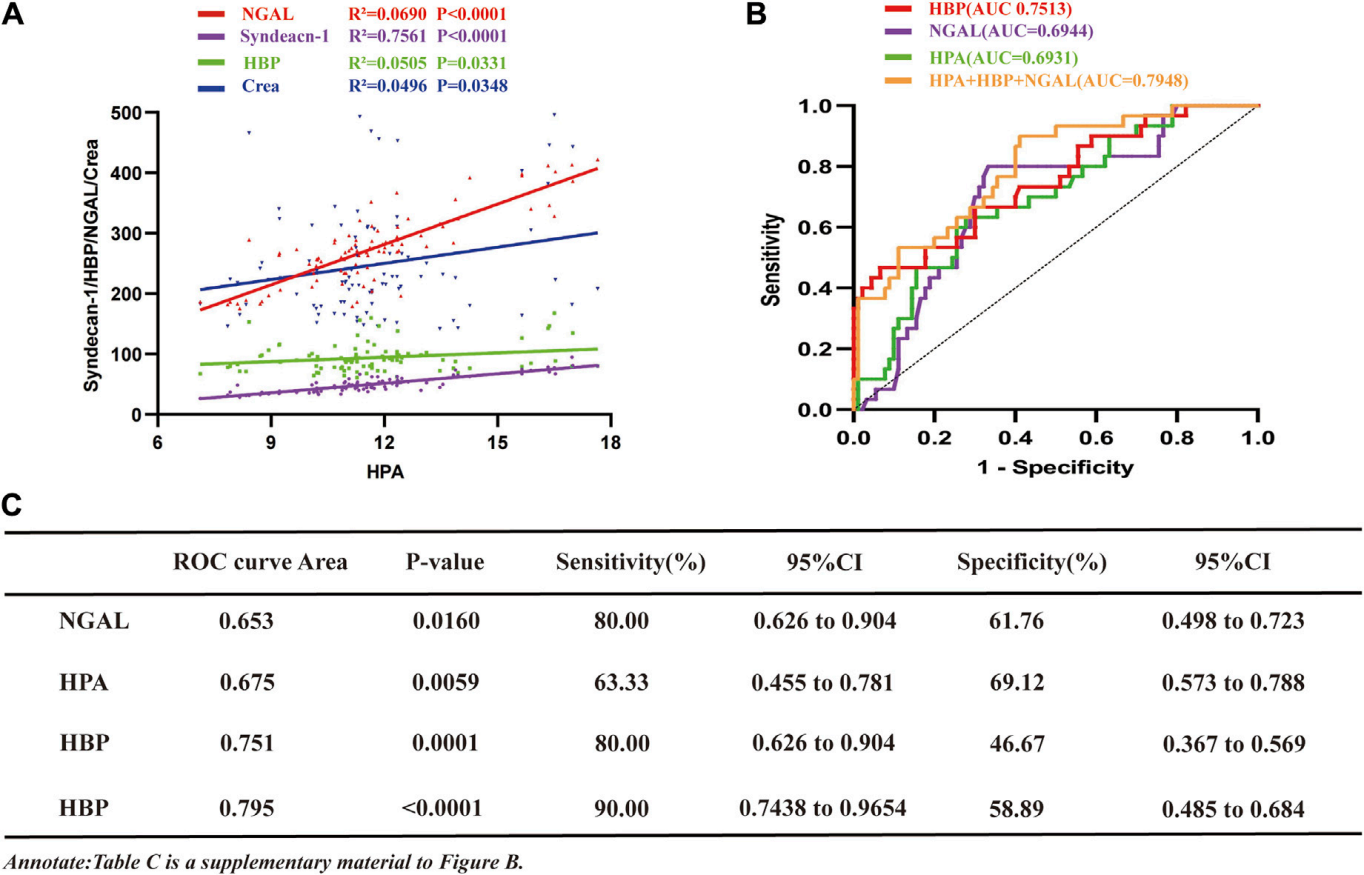
The study investigates the associations of HPA with NGAL, HBP, Creatinine, and Syndecan-1 in sepsis, analyzes ROC curves for HPA, NGAL, and HBP in SA-AKI. **(A)** Correlation curve depicting the relationship between HPA and NGAL, HBP, syndecan-1 and creatinine in sepsis patients (NGAL, R^2^ = 0.0690, *p* < 0.001; syndecan-1, R^2^ = 0.7561, *p* < 0.001; HBP, R^2^ = 0.0505, *p* = 0.0331; NGAL, R^2^ = 0.0496, *p* = 0.0348). **(B)** ROC curves comparing NGAL, HBP, HPA and their combination for the diagnosis of SA-AKI and sepsis without AKI (sepsis without AKI group, *n* = 30, SA-AKI group, *n* = 90; HBP, AUC = 0.7513; NGAL, AUC = 0.6944; HPA, AUC = 0.6931; NGAL + HBP + HPA, AUC = 0.7948). **(C)** ROC values, *p*-values, sensitivity, specificity results, and their confidence intervals for **(B)**. HPA, heparanase; NGAL, neutrophil gelatinase-associated lipocalin; HBP, heparin-binding protein; AKI, acute kidney injury; SA-AKI, sepsis-associated acute kidney injury; ROC, receiver operating characteristic; AUC, area under curve; LMWH low molecular weight heparin; CI, confidence intervals.

To evaluate the relationship between HPA concentrations in the studied population and NGAL, HBP, syndecan-1, and creatinine, a correlation analysis was conducted (Figure 2A). The findings revealed a positive correlation between HPA and NGAL (R^2^ = 0.0690), HBP (R^2^ = 0.0505), syndecan-1 (R^2^ = 0.7561) and creatinine (R^2^ = 0.0496).

#### 3.2.2 Diagnostic value of HPA, HBP, NGAL and their combination in SA-AKI

To assess the comparative predictive efficacy of HPA, HBP, NGAL and their combination in the diagnosis of SA-AKI, ROC curve analysis was conducted (Figure 2B). The results demonstrated that the area under the curve (AUC) for SA-AKI was the largest for the combination (0.7948), with a sensitivity of 90% and specificity of 58.89% (Figure 2C). HBP, HPA and NGAL had AUC values of 0.7513, 0.6944 and 0.6931, respectively. Notably, NGAL exhibited the highest sensitivity (80%), followed by HPA (63.33%), both surpassing HBP (46.67%) (Figure 2C). However, HBP demonstrated superior specificity (93.33%), while NGAL and HPA had specificities of 66.67% and 72.22%, respectively (Figure 2C).

### 3.3 Mendelian randomization analysis

A causal link between HBF and renal tubule injury has been confirmed. To elucidate the relationship between HBP and kidney injury, we conducted MR Analysis. Adhering to the established screening criteria for IVs, we identified 32 SNPs to analyzing the causal link between HBF and renal tubular injury (Supplementary Table S1). Importantly, the F statistic for all IVs exceeded 10, indicating the absence of weak IV bias.

**FIGURE 3:**
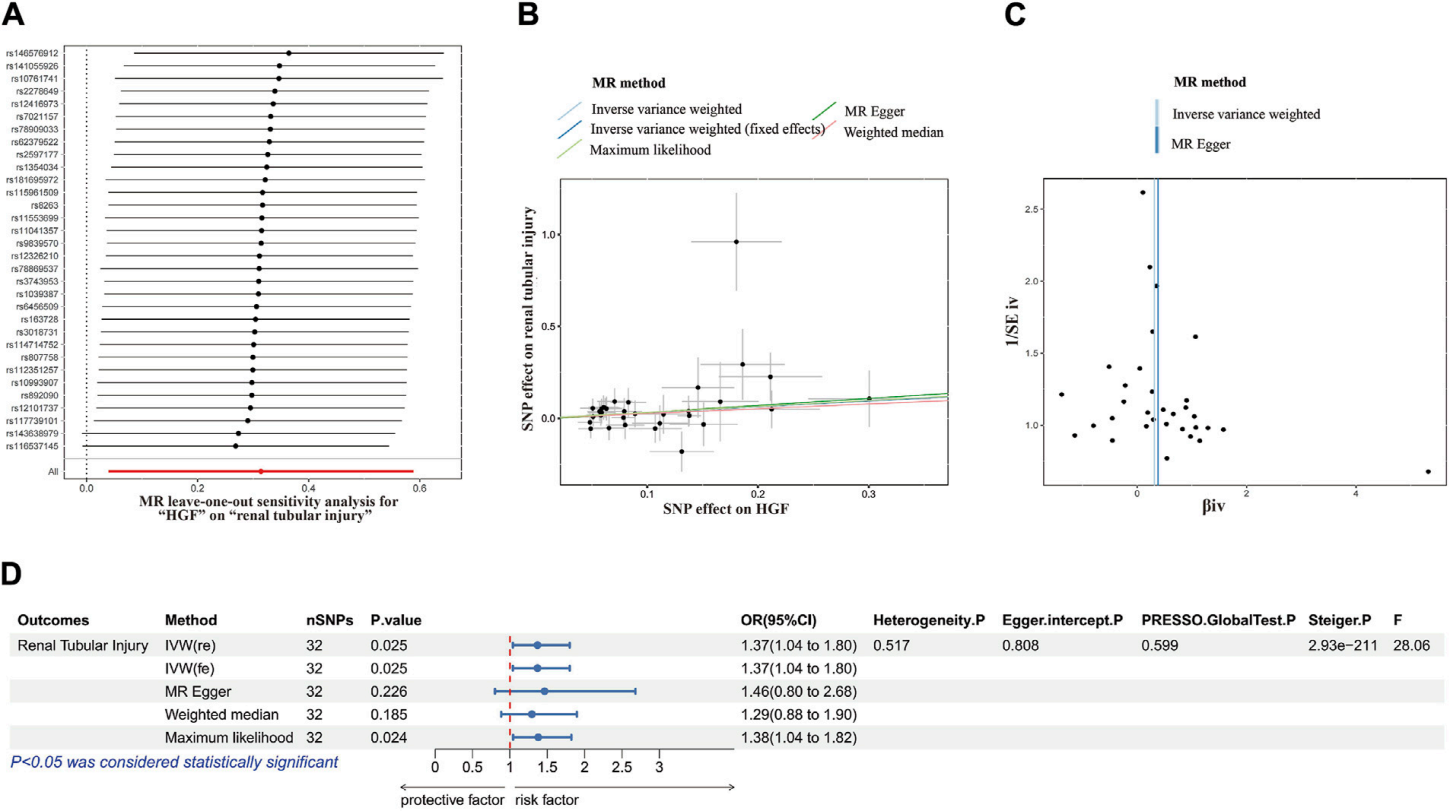
MR analysis correlation analysis plot. **(A)** Forest plots for the MR leave-one-out analysis of the significant IVW estimates. **(B)** Scatterplots of SNPs’ effects on HGF versus their effects on renal tubular injury. **(C)** Funnel plots of the association between HGF and renal tubular injury. **(D)** The forest map includes a MR analysis of HGF on renal tubular injury and regression results of sensitivity analysis, Steiger test, and F-statistic. MR, Mendelian randomization; SNP, single nucleotide polymorphisms; HGF, heparin-binding growth factors; IVW, inverse variance weighting.

Consistently across the IVW method, MR-Egger method, weighted median method and maximum likelihood method in MR analysis, all demonstrated consistent outcomes (Figures 3A–C). Specifically, in the IVW analysis, a causal association was established between HBF and tubular injury (OR value: 1.369, 95%CI: 1.040–1.801, *p* = 0.024). This implies a positive correlation, indicating that the risk of tubular injury rose by approximately 36.8% for every one standard deviation increase in HBF.

In subsequent sensitivity analyses, Cochrane’s Q test revealed no significant heterogeneity (*p* > 0.05). Various methods, including MR-Egger regression, MR-PRESSO, and the funnel plot method, found no evidence of pleiotropy (*p* > 0.05). Additionally, leave-one-out analysis further excluded any causal relationship between single SNP-driven exposure and outcome. The Steiger direction test confirmed that all Steiger directiaons were TRUE, with a *p*-value <0.05, indicating the absence of reverse causality in the results. Our forest plot, incorporating these findings, illustrated the effect size between HBF and tubular damage (Figure 3D).

### 3.4 LMWH improved renal function in patients with SA-AKI

To assess the impact of LMWH on renal function in SA-AKI patients, we categorized SA-AKI patients into control and LMWH treatment groups, based on the administration of LMWH.

#### 3.4.1 Baseline data of control group and LMWH group

The two groups exhibited a good match, with no statistically significant differences between them except for BMI (Table 2).

**TABLE 2:** Characteristics of the patients at baseline. (Mean ± SD).

Parameter	Control group (*n* = 45)	LMWH group (*n* = 45)	*p*-value
Age (year)	61.51 ± 16.41	66.36 ± 13.83	0.155
Sex-no. (%)			>0.9999
Male	30 (66.67)	31 (68.89)	
Female	15 (33.33)	14 (31.11)	
BMI (kg/m^2^)	24.66 ± 4.57	22.97 ± 3.34	0.048
APACHE II score^a^	29.58 ± 4.78	29.76 ± 4.48	0.856
SOFA score^b^	9.22 ± 1.93	9.60 ± 1.74	0.332
MAP (mmHg)	114.42 ± 89.06	93.21 ± 19.78	0.170
Serum creatinine (umol/L)	246.16 ± 14.05	248.53 ± 12.66	0.900
Plasma HPA (ng/mL)	11.53 ± 2.44	11.85 ± 2.00	0.291
Plasma HBP (ng/mL)	92.81 ± 24.87	95.08 ± 22.80	0.362
Oliguria-no. (%)	10 (22.22)	12 (26.67)	ns
Anuria-no. (%)	5 (11.11)	4 (8.89)	ns
Staging of AKI—no. (%)			0.2500
Stage 1	17 (37.78)	11 (24.44)	
Stage 2	19 (42.22)	21 (46.67)	
Stage 3	9 (20.00)	13 (28.89)	

Abbreviations: LMWH, low molecular weight heparin; AKI, acute kidney injury; BMI, body mass index; MAP, mean arterial pressure; HPA, heparanase; HBP, heparin-binding protein; COPD, chronic obstructive pulmonary disease.

^a^ The Acute Physiology and Chronic Health Evaluation (APACHE) II, score ranges from 0 to 71, with higher scores indicating greater risk of hospital death.

^b^ The Sequential Organ Failure Assessment (SOFA) score ranges from 0 to 24, with higher scores indicating greater severity of organ dysfunction. *p* < 0.05 was statistically significant.

#### 3.4.2 LMWH improved renal function in patients with SA-AKI

**FIGURE 4:**
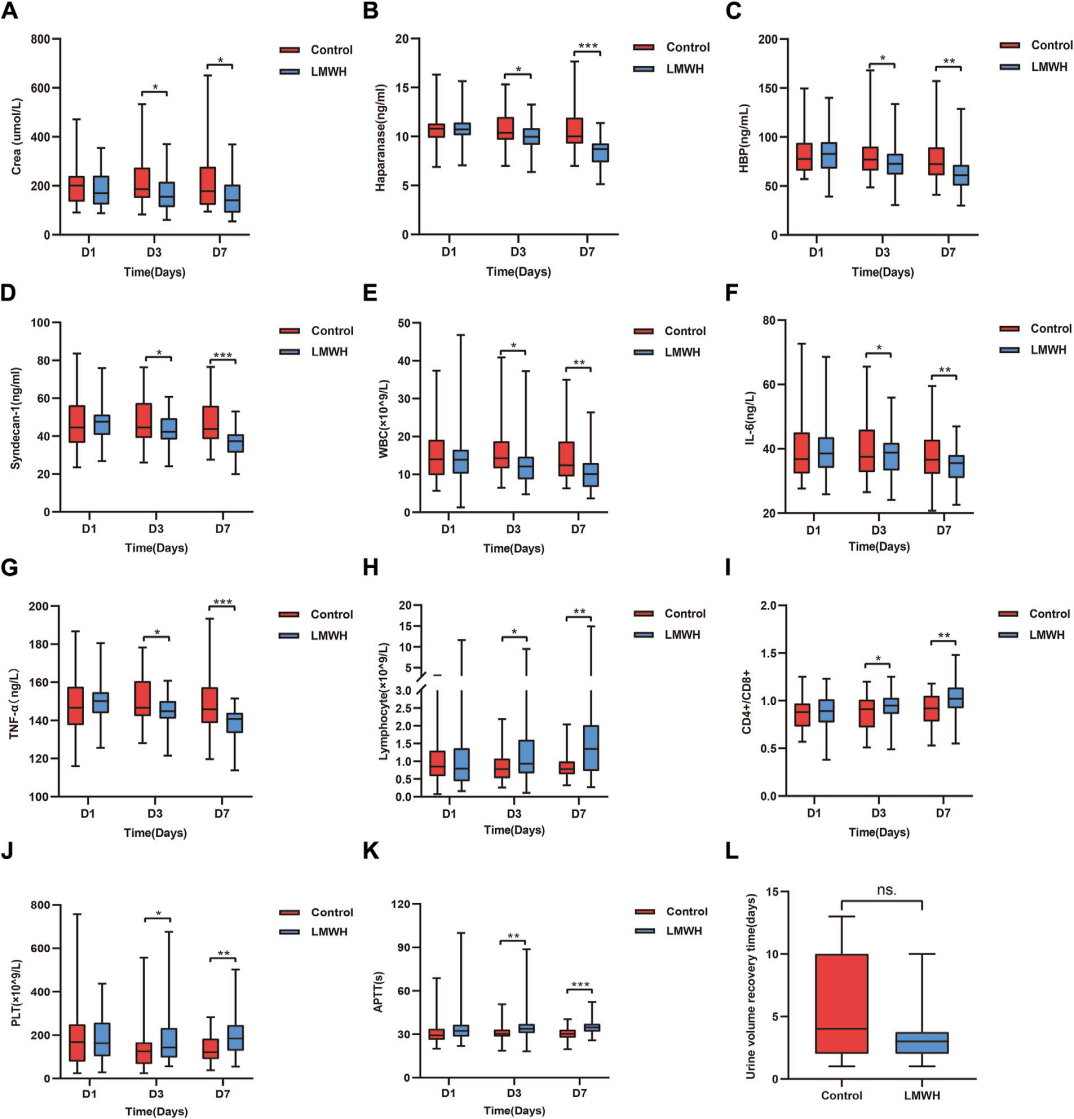
Results of the control and LMWH groups after LMWH calcium treatment on the 1st, 3rd, and 7th days. **(A)** Concentration of serum Crea after treatment in the control and LMWH groups. **(B)** Concentration of plasma HPA after treatment in the control and LMWH groups. **(C)** Concentration of plasma HBP treatment in the control and LMWH groups. **(D)** Concentration of plasma syndecan-1 after treatment in the control and LMWH groups. **(E)** Concentration of blood WBC after treatment in the control and LMWH groups. **(F)** Concentration of plasma IL-6 after treatment. **(G)** Concentration of plasma TNF-α after treatment. **(H)** Levels of blood lymphocyte count after treatment in the control and LMWH groups. **(I)** Levels of blood CD4+/CD8+ after treatment in the control and LMWH groups.**(J)** Levels of blood PLT count after treatment in the control and LMWH groups. **(K)** Levels of APTT after treatment in the control and LMWH groups. **(L)** Urine volume recovery time in patients with oliguria and anuria. Statistically significant differences are indicated as *p* < 0.05. (*p < 0.05, **p < 0.05, ***p < 0.001.) IL-6, interleukin-6; TNF-α, tumour necrosis factor-α; APTT, plasma activated partial thromboplastin time; PLT, platelet; HPA, heparanase; HBP, heparin-binding protein; WBC, white blood cells; Crea, creatinine; LMWH, low molecular weight heparin.

Compared to the control group, the LMWH group showed a significant reduction in serum creatinine levels on the 3rd and 7th day (*p* < 0.05). No significant differences were observed between the two groups on the 1st day (Figure 4A). Additionally, LMWH treatment facilitated a quicker recovery of urine volume in patients with anuria and oliguria (*p* < 0.05) (Figure 4L). Renal function grades improved in both groups on the 7th day after treatment, with notable intergroup differences (Table 3).

**TABLE 3:** The stage of AKI.

	Normal-n (%)		AKI 1-n (%)		AKI 2-n (%)		AKI 3-n (%)		Z value	*p*-Value
	Control	LMWH	Control	LMWH	Control	LMWH	Control	LMWH		
D1	2 (4.44)	4 (8.89)	20 (44.44)	21 (46.67)	14 (31.11)	13 (28.89)	9 (20.00)	7 (15.56)	−0.829	0.407
D3	3 (6.67)	8 (17.78)	19 (42.22)	17 (37.78)	11 (24.44)	13 (28.89)	10 (22.22)	6 (13.33)	−0.534	0.224
D7	7 (15.56)	15 (33.33)	14 (31.11)	13 (28.89)	7 (15.56)	6 (13.33)	8 (17.78)	4 (8.89)	−1.972	0.049

*p* < 0.05 was statistically significant.

#### 3.4.3 LMWH resulted in a reduction in plasma concentrations of HPA, HBP, and syndecan-1 on the 3rd and 7th day within the LMWH treatment group

Compared to the control group, the LMWH group exhibited a notable decrease in HPA, HBP and syndecan-1 concentrations on the 3rd and 7th days (*p* < 0.05 and *p* < 0.001, respectively). No significant differences were observed between the two groups on the 1st day (Figures 4B–D).

#### 3.4.4 LMWH attenuated inflammation, improved immune function on the 3rd and 7th day

Compared to the control group, the LMWH group displayed reduced levels of WBC, IL-6 and tumor necrosis factor (TNF)-a on the 3rd day (*p* < 0.05), with inflammation significantly decreased on the 7th day (*p* < 0.01 vs. *p* < 0.001) (Figures 4E–G). Regarding immunity, the LMWH group exhibited an increased lymphocyte count and CD4+/CD8+ ratio on the 3rd and 7th days (*p* < 0.05, *p* < 0.01). No significant differences were observed between the two groups on the 1st day (Figures 4H, I).

#### 3.4.5 LMWH affected blood clotting in SA-AKI patients

In terms of blood clotting, the LMWH group demonstrated prolonged activated partial thromboplastin time (APTT) on the 3rd and 7th days (*p* < 0.05, *p* < 0.001, respectively), alongside a significant increase in platelet count on the 3rd and 7th days (*p* < 0.05 vs. *p* < 0.01). Once more, there were no significant differences between the two groups on the 1st day (Figures 4J, K).

### 3.5 Clinical prognosis

The primary outcome assessed was renal function, with secondary outcomes including 28-day mortality, 90-day survival rate, the number of patients receiving RRT, and the median length of ICU stay. The findings revealed no significant difference in 28-day mortality or length of ICU stay between the control and LMWH groups (Figure 2C). Moreover, there were no significant differences in 90-day survival, the number of patients receiving RRT, or adverse reactions between the two groups (Table 4).

**TABLE 4:** Outcomes.

Parameters	Control	LMWH	*p*-Value
Primary Outcome			
Serum creatinine (umol/L)	209.63 ± 117.85	154.43 ± 83.50	0.007
Secondary outcomes			
Death at 28 days—no. (%)	31 (68.89)	25 (55.56)	0.2769
Survival at 90 days—no. (%)	13 (28.89)	16 (35.56)	0.6523
Patients who received renal-replacement therapy—no. (%)	8 (17.70)	9 (20.0)	ns
Median length of ICU stay (IQR)—days			ns
Survivors	30 (28-36)	30 (16.25–30)	
Nonsurvivors	13.5 (6.5–17)	10 (6.5–17.5)	
Adverse events that occurred during the trial—no. (%)			ns
Gastrointestinal complication	15 (33.3)	12 (26.7)	
Thrombotic or embolic complication	3 (6.67)	4 (20.0)	
Severe cardiac-rhythm disorder	2 (6.0)	1 (5.0)	
Severe bleeding event	0 (0)	0 (0)	

## 4 Discussion

Our study primarily demonstrated a significant association between elevated plasma HPA and HBP levels and the presence as well as progression of AKI in sepsis, thereby indicating that HPA and HBP exert a pivotal role in the pathophysiology of SA-AKI, which aligns with previous research findings (Fisher et al., 2017; Poston and Koyner, 2019; Liu et al., 2023). Clearance correlation analysis revealed a positive association between HPA and NGAL, HBP, creatinine, and syndecan-1. Consequently, we integrated NGAL, HBP, and HPA to conduct ROC curve analysis and observed that this combination exhibited superior predictive ability for early SA-AKI diagnosis. Additionally, we found that LMWH effectively inhibited HPA and HBP-induced inflammation in renal cells, indicating the potential of targeting HPA and HBP for SA-AKI treatment. Furthermore, through MR analysis, we established a direct association between HBF and renal tubular injury, consistent with findings from clinical observational studies. Finally, we hypothesized that the potential mechanism underlying the beneficial effects of LMWH in ameliorating SA-AKI may involve attenuation of HPA activity, inhibition of ECM degradation, and subsequent reduction in FGF release, thereby leading to improved renal function in SA-AKI.

Previous *in vitro* and *in vivo* studies have shown increased activation of the HPA axis in glomerular and interstitial vascular endothelial cells during sepsis. However, renal function improved with the use of HPA inhibitors (Chen et al., 2015). Based on these findings, we hypothesised that HPA inhibitors may improve renal function in patients with SA-AKI, suggesting HPA may be a therapeutic target for SA-AKI. Consequently, we categorized SA-AKI patients into control and LMWH groups to investigate the impact of LMWH on renal function and clinical prognosis by modulating HPA activity. Our study represented a pioneering investigation into elucidate the role of HPA in patients with SA-AKI, unequivocally demonstrating that pharmacological inhibition of HPA exerts a beneficial effect on renal function in SA-AKI.

The findings from murine sepsis models have demonstrated that HPA promotes the shedding of endothelial glycocalyx, leading to vascular leakage and decreased blood volume, consequently contributing to increased sepsis related mortality (Song et al., 2017). Glycocalyces, mainly composed of HSPGs and glycosaminoglycans, cover the surface of the endothelium and play a crucial role in regulating vascular permeability, coagulation, platelet and white blood cell adhesion, as well as anti-inflammatory and antioxidant processes (Sieve et al., 2018; Lupu et al., 2020). Syndecan-1 serves as a biomarker of glycocalyx degradation (Rehm et al., 2007) and is released into the blood under stressful conditions such as AKI, chronic kidney disease, and cardiovascular disease (Puskarich et al., 2016). Consistent with our study, inhibiting HPA reduced syndecan-1 levels.

In addition, we observed that HPA can exert an influence on inflammation, immune response, and coagulation function in patients with SA-AKI. During sepsis, HPA is activated by pathogen-associated molecular patterns and inflammatory cytokines. In our study, LMWH was employed, which exerted anti-inflammatory effects by inhibiting HPA activity, thereby safeguarding renal function and ameliorating renal inflammation, consistent with previous studies (Achour et al., 2016; Mayfosh et al., 2019). Evidence also indicates that HPA mediates interactions among various immune cell types, including T cells, B cells, natural killer cells, macrophages, neutrophils, and dendritic cells (Mayfosh et al., 2019). LMWH has the capability to prevent activation of the complement system, and the inhibition of C5a prevents the release of inflammatory and prothrombotic molecules such as TNF-α and tissue factor (Rao et al., 1993). In our study, we observed improved immune function in the LMWH group compared with the control group, suggesting that LMWH may also enhance renal immune function in patients with SA-AKI. The anticoagulant effect of LMWH is related to its binding to antithrombin III, which inhibits coagulation and platelet aggregation, leading to alterations in APTT. Consistent with these mechanisms, we observed that APTT was significantly prolonged in the LMWH group. Previous studies have reported that LMWH has anti-inflammatory properties independent of its anticoagulant effects (Luan et al., 2014). With the progress of treatment, the platelet count gradually recovered. The platelet level of patients with SA-AKI in the LMWH group was significantly increased, and the coagulation function was improved. The effects of LMWH on platelet recovery also could be due to reduced platelets bound to the vessel wall.

In terms of clinical outcomes, no significant differences were noted between the two groups concerning 28-day mortality, 90-day survival, and duration of ICU stay. Several plausible explanations underlie these findings. Firstly, patients with SA-AKI typically present with a constellation of comorbidities, and the length of their hospitalization is subject to a multitude of influencing factors. Secondly, the current usage of LMWH primarily as an anticoagulant may not wield sufficient impact on mortality and survival rates in SA-AKI patients. Moreover, the LMWH employed in our investigation lacked specificity as an HPA inhibitor, as safe clinical-grade HPA inhibitors remain elusive. Furthermore, owing to the constrained sample size in our study, the potential impact of LMWH on the mortality and survival rates of patients with SA-AKI by inhibiting HPA activity may not have been comprehensively elucidated. Nonetheless, our study unearthed noteworthy disparities in SA-AKI grade post-treatment between the two cohorts, with HPA inhibitor utilization correlating with diminished requirements for RRT and reduced hospitalization costs, thus alleviating the financial burden on patients. Notably, to the best of our knowledge, such findings have not been previously documented in SA-AKI patients. Our study disclosed a 28 days mortality rate of approximately 70% among patients with an average SOFA score of 9, markedly surpassing previously reported rates (Raith et al., 2017). The patients included in this study were generally older and frequently presented with severe infections. Owing to economic constraints, many patients were discharged upon symptomatic improvement, albeit without complete disease control, potentially contributing to escalated mortality rates. Additionally, the study sample was robust but exhibited inherent biases, alongside elevated mortality rates.

In conclusion, our study supports previous findings indicating a higher incidence of SA-AKI compared to septic patients without AKI. We have identified plasma HPA as a promising biomarker for early diagnosis of SA-AKI. Furthermore, our findings suggest that inhibiting HPA could potentially improve renal function in patients with SA-AKI. The correlation we observed between HPA levels and biomarkers of renal damage, including NGAL, HBP, creatinine, and syndecan-1, further strengthens the link between HPA and renal structural injury. In addition, our study highlights the potential of LMWH in reducing HPA levels, thereby enhancing renal function, mitigating inflammation and immune response, and modulating coagulation in SA-AKI patients. Although no significant differences in clinical outcomes were observed, the improvements in renal function grading, reduced need for RRT, and lower hospitalization costs among SA-AKI patients suggest potential advantages of HPA inhibition in SA-AKI management. However, comprehensive investigations with larger cohorts and specific HPA inhibitors are warranted to fully elucidate the therapeutic efficacy of targeting HPA in SA-AKI treatment.

Our study possesses several notable strengths:

1. Clinical Relevance: Unlike previous investigations primarily conducted on cellular and animal models, our study is pioneering in directly observing the effects of inhibiting HPA on renal function in SA-AKI patients, thus enhancing its clinical relevance.
2. Elaboration of Causal Links: We have further substantiated the causal relationship between HBP and renal tubular injury in septic kidneys. Notably, our findings revealed that the combined assessment of HPA, HBP, and NGAL offers superior predictive value for early SA-AKI diagnosis, adding depth to our understanding of its pathogenesis.
3. Insights into LMWH Mechanism: Our study provides insights into a potential mechanism underlying the improvement of renal function in SA-AKI patients with LMWH administration. Specifically, we propose that LMWH’s efficacy may be attributed, at least in part, to its inhibition of HPA activity, leading to reduced ECM degradation. This cascade results in diminished bFGF release, ultimately contributing to enhanced renal function.

Our study also exhibits certain limitations:

1. Lack of Urine Samples for HPA Detection: HPA emanates from various sources, with platelets being notably significant (Eustes et al., 2021). Consequently, the kidney may not singularly serve as the origin of HPA, rendering plasma-based measurements insufficient for an accurate depiction of renal HPA levels. Due to instances where patients presenting with oliguria or anuria upon admission, the acquisition of urine specimens was unattainable, necessitating our reliance on plasma HPA assessments predominantly. Additionally, assays for syndecan-1 and NGAL were conducted on plasma samples rather than urine, potentially compromising the sensitivity and specificity of HPA in the diagnosis of SA-AKI. Furthermore, the assessment of HPA enzyme activity, typically involving complex techniques, was not performed in our study.
2. Single-Center Design and Limited Sample Size: The study’s single-center design and constrained sample size pose potential biases, especially considering the presence of various complications in enrolled patients. This limitation could impact the significance of disparities noted in immune function, coagulation function, and mortality rates between the control and LMWH groups, thereby potentially restricting the generalizability of our findings. In addition, the sensitivity and specificity reported in ROC curve analysis differ from previous studies, underscoring the need for enlarging sample size and conducting multicenter studies to validate our results.
3. Representation of Population Ancestry: Our study encountered challenges concerning the representation of population ancestry. The MR analysis primarily involved individuals of European descent, while the cohort study centered on an Asian population. Although our findings aligning consistently with previous real-world studies (Carter et al., 2003; Lee et al., 2012; Higashi et al., 2020; Pajenda et al., 2020), we recommend further validation in multi-center, multi-ancestry populations to enhance the broader relevance of our conclusions.

## 5 Conclusion

LMWH can improve renal function in critically ill patients with SA-AKI, and the potential mechanism may be that LMWH reduces the release of HBP by inhibiting the activity of HPA, as suggested by potential mechanisms. Moreover, MR Analysis underscores a corresponding causal link. These results also highlight the necessity for future randomized controlled trials to corroborate these observations.

## Data Availability

The original contributions presented in the study are included in the article/Supplementary Material, further inquiries can be directed to the corresponding author.
